# Chain Mediation of Psychological Capital and Burnout Between Nurses’ Artificial Intelligence Literacy and Occupational Resilience: A Cross‐Sectional Study

**DOI:** 10.1155/jonm/8230166

**Published:** 2026-08-27

**Authors:** Qingzhu Qin, Wenjing Sun, Hengyu Hu, Yixiu Du, Cancan Chen

**Affiliations:** ^1^ Department of Cardiology, Henan Provincial People’s Hospital, Zhengzhou, Henan, China, hnsrmyy.net; ^2^ Department of Geriatrics, Henan Provincial People’s Hospital, Zhengzhou, Henan, China, hnsrmyy.net; ^3^ School of Nursing, Henan University of Chinese Medicine, Zhengzhou, Henan, China, hactcm.edu.cn

**Keywords:** artificial intelligence literacy, burnout, management, nurses, occupational resilience, psychological capital

## Abstract

**Background:**

AI literacy is increasingly recognized as a core competency that enables nurses to improve work efficiency and reduce workload. Therefore, enhancing AI literacy represents a promising strategy to strengthen occupational resilience. However, current research has not yet elucidated the direct relationship and underlying mechanisms between nurses’ AI literacy and occupational resilience.

**Aim:**

To investigate the impact of nurses’ artificial intelligence literacy on their occupational resilience and examine the chain mediating effects of psychological capital and burnout.

**Methods:**

Data were collected from December 2025 to January 2026 using self‐report electronic questionnaires completed by nurses in 10 medical institutions across China. Descriptive analysis, correlation analysis, and chain mediation analysis were performed on all study variables.

**Results:**

Correlation analysis showed that nurses’ artificial intelligence literacy was significantly positively correlated with occupational resilience (*r* = 0.351, *p* < 0.001) and psychological capital (*r* = 0.356, *p* < 0.001) and significantly negatively correlated with burnout (*r* = −0.130, *p* < 0.05). Bootstrap analysis revealed that psychological capital served as the primary mediating pathway (effect = 0.377, 95% CI = [0.283, 0.475]), and a sequential chain mediation via psychological capital and burnout also existed between artificial intelligence literacy and occupational resilience (effect = 0.036, 95% CI = [0.019, 0.057]).

**Conclusion:**

Artificial intelligence literacy enhances occupational resilience through the chain mediation effects of psychological capital and burnout. Occupational resilience among nurses can be strengthened by implementing artificial intelligence skills training and psychological capital interventions for nursing staff.

**Implication for Nursing Management:**

Nursing managers should implement hierarchical artificial intelligence training, regularly assess nurses’ psychological capital and burnout and offer targeted interventions. Meanwhile, healthcare institutions should upgrade digital infrastructure, adopt artificial intelligence–enabled nursing technologies, and establish guidelines for intelligent nursing applications to systematically enhance nurses’ occupational resilience.

## 1. Introduction

As a core component of the global healthcare workforce, nurses have long faced heavy workloads, strained nurse–patient interactions, and barriers to career advancement, leading to substantial occupational stress. Occupational resilience—the capacity to cope with work‐related stress, obstacles, or adversity—plays a pivotal role in shaping work attitudes, decisions, and behaviors [1]. Strengthening nurses’ occupational resilience not only mitigates negative consequences of workplace stress, including burnout, compassion fatigue, and turnover intentions, but also improves patient outcomes [2]. Enhancing nurses’ professional competence and supporting their career advancement have been validated as effective strategies for strengthening occupational resilience [3, 4]. With the rapid development of digital health care, artificial intelligence (AI) has been widely integrated into modern nursing practice, including clinical decision support, patient management, and workflow optimization, thereby reducing nurses’ workload [5]. Adequate AI literacy—the ability to understand, apply, and evaluate AI technologies—enables nurses to adapt to the digitalized nursing environment and may serve as a vital pathway to improve their occupational resilience [6].

Few studies have explored the potential mental health benefits of AI literacy. Existing scoping reviews have shown that AI literacy contributes to mitigating depression and enhancing psychological well‐being and resilience [7]. Although theoretical reviews have proposed that AI literacy may alleviate job burnout among nurses [8], these claims lack empirical support. To date, empirical evidence on the effects of AI literacy on nurses’ psychological states and occupational adaptability remains scarce [5]. Specifically, the direct association between nurses’ AI literacy and occupational resilience has not been empirically established, and the underlying mediating mechanisms of this relationship remain underexplored.

Integrating the Conservation of Resources (COR) theory [9] and the Job Demands–Resources (JD‐R) model [10], this study empirically examines the association between nurses’ AI literacy and occupational resilience and further explores the mediating roles of key psychological factors to elucidate the underlying mechanism through which AI literacy shapes occupational resilience. COR theory explains individual resource dynamics, whereas the JD‐R model elucidates how work characteristics shape occupational well‐being. According to COR theory, resource loss leads to psychological distress, whereas resource accumulation enhances occupational resilience [9]. The JD‐R model posits that job resources buffer the negative impact of job demands and foster motivation [10]. In this study, nurses’ AI literacy is defined as a digital job resource under the JD‐R model and a practical occupational resource under COR theory. In clinical practice, nurses with high AI literacy can use intelligent systems to automatically generate nursing records, conduct patient risk screening, and optimize workflows. These digital tools reduce repetitive work, facilitate psychological capital accumulation, and mitigate rapid resource depletion [5]. Conversely, nurses with limited AI literacy adapt poorly to digital nursing practices, deplete resources faster, and face higher burnout and lower occupational resilience. Based on the above, the following hypotheses were proposed:

 H1. AI literacy positively predicts psychological capital.
 H2. AI literacy negatively predicts job burnout.
 H3. AI literacy enhances occupational resilience through the chain mediating effects of psychological capital and burnout.

By integrating AI literacy into the research framework of occupational resilience, this study differs from conventional studies that focus solely on individual psychology and work environments. The findings provide practical implications for nursing managers to implement strategies, such as skill training and psychological interventions, thereby strengthening nurses’ occupational resilience and maintaining a stable, high‐quality nursing workforce.

## 2. Materials and Methods

### 2.1. Study Design

A multicenter cross‐sectional design was used in this study. Self‐report data were collected from nurses working in China via an online survey. The manuscript adheres to the STROBE checklist for cross‐sectional studies.

### 2.2. Study Setting and Sampling

From December 2025 to January 2026, nurses were recruited using convenience sampling. Participants worked in 10 hospitals across three Chinese provinces: Henan, Zhejiang, and Beijing. The inclusion criterion required that all participants hold a valid nurse practicing certificate. Nurses who were absent due to sick leave or maternity leave were excluded.

The G∗Power 3.1 program [11] was used to calculate the required sample size. With a significance level of 0.05, a power of 0.95, an effect size of 0.15, and 10 predictors, a minimum sample of 172 participants was needed for regression analysis. To account for invalid questionnaires, the sample size was increased by 20%. Thus, the recommended minimum size was 207.

### 2.3. Variables and Instruments

#### 2.3.1. Demographic Information

Using a demographic form created by the researchers, the following nurses’ characteristics were collected: gender, age, marital status, education, professional title, occupational role, and AI‐related training participation.

#### 2.3.2. AI Literacy

The Artificial Intelligence Literacy Scale (AILS), developed by Chinese scholars, was directly adopted in this study [12]. It is a 12‐item instrument with a 7‐point Likert‐type scale ranging from “*strongly disagree*” to “*strongly agree*.” The scale comprises four dimensions: awareness, practical application, evaluative judgment, and ethical reasoning. The total possible score ranges from 12 to 84, with higher scores indicating greater individual AI literacy. This scale has established construct validity reported in the original development study. The scale’s Cronbach’s α was 0.83, and in the current study, it was 0.84.

#### 2.3.3. Psychological Capital

Psychological capital was measured using the Psychological Capital Questionnaire (PCQ‐24), which was directly adopted in the present study [13]. It comprises 24 items across four dimensions: self‐efficacy, resilience, hope, and optimism. All items were assessed using a 6‐point Likert scale ranging from 1 (*strongly disagree*) to 6 (*strongly agree*). A higher total score indicates a higher level of psychological capital. The scale shows favorable construct validity, supported by exploratory and confirmatory factor analyses. The scale’s Cronbach’s α was 0.90, and in the current study, it was 0.93.

#### 2.3.4. Burnout

Burnout was evaluated using the Chinese version of the Maslach Burnout Inventory General Survey (MBI‐GS), which was originally developed by Maslach and Jackson [14] and translated by Li and Liu [15]. This 22‐item self‐report scale includes three dimensions: emotional exhaustion, depersonalization, and personal accomplishment. Emotional exhaustion and depersonalization items were scored on a 0–6 Likert scale (0 = *never*, 6 = *every day*), whereas personal accomplishment items were reverse‐scored and renamed as reduced personal accomplishment in the analysis. The total score ranged from 0 to 132, with higher scores indicating greater burnout severity. The Chinese version demonstrates satisfactory content validity and construct validity. The scale’s Cronbach’s α was 0.94, and in the current study, it was 0.86.

#### 2.3.5. Occupational Resilience

Occupational resilience was measured using the Career Motivation Inventory (CMI). This scale is specifically designed to evaluate occupational resilience in the Chinese workplace context and was directly adopted in the present study [16]. The scale contains 25 items and comprises three dimensions: affective, cognitive guidance, and behavioral guidance. Sample items include “When facing occupational difficulties, I am able to develop targeted coping plans” and “I improve myself through overcoming occupational setbacks.” All items were positively scored on a 5‐point Likert scale, with responses ranging from 1 (*strongly disagree*) to 5 (*strongly agree*). The total achievable score ranges from 25 to 125, with higher scores indicating greater occupational resilience. The scale has been verified to possess good construct validity through exploratory and confirmatory factor analyses. The original scale demonstrated good reliability, with Cronbach’s α values ranging from 0.71 to 0.88 [16]. In the present sample, the overall Cronbach’s α of the scale was 0.97.

### 2.4. Data Collection

Data collection for this study was conducted via Wenjuanxing (https://www.wjx.cn/), a widely recognized, reliable, and efficient electronic questionnaire tool within the Chinese academic community [17]. During questionnaire development, the research team carefully reviewed and revised the instrument, refining the title, introduction, core objectives, practical implications, research methods, and completion instructions. Following the first draft, a pilot survey was conducted on Wenjuanxing to test feasibility. The results indicated the need for further optimization, which enhanced the scientific rigor and validity of the final version. After the questionnaire was finalized, the principal investigator informed relevant hospital administrators of the study’s objectives, procedures, and expected outcomes. Upon approval, the questionnaire link and QR code were shared with nursing teams across departments via WeChat, a widely used social media application in China. All participating nurses completed the questionnaire voluntarily. To ensure data quality, all items were mandatory, and only one submission per IP address was allowed. For confidentiality, collected data were encrypted and accessible only to core research team members. For data screening, questionnaires with identical answers to 15 consecutive items or a total response time of less than 300 s were deemed invalid and excluded.

### 2.5. Data Analysis

IBM SPSS Statistics 26.0 was used to perform all data analyses in this study. Harman’s single‐factor test was performed to assess common method bias. Descriptive statistics were computed for demographic variables and for the total scores of the AILS, PCQ‐24, MBI‐GS, and CMI. Independent‐samples *t*‐tests or one‐way analysis of variance was performed to compare CMI scores across demographic groups. Variables that showed significant differences were included as covariates in the multiple mediation models. Pearson’s correlation analysis was conducted to examine the correlations among the AILS, PCQ‐24, MBI‐GS, and CMI. Hayes’ PROCESS macro (Model 6) for SPSS was used to test the chain mediation model. A bias‐corrected bootstrap method with 5000 resamples was adopted. Standardized regression coefficients and 95% confidence interval (CI) for the direct, indirect, and total effects were reported, with a mediating effect considered significant if the 95% CI did not include zero. The significance level for all statistical tests was 0.05 (two‐sided).

### 2.6. Ethical Considerations

Ethics approval was obtained from the Henan Provincial People’s Hospital (approval number: 2024114), and all participants provided informed consent. In the study invitation letter, participants were informed that completing the survey questionnaire would be regarded as their voluntary informed consent. The study explicitly stated that participation was entirely voluntary and that participants could withdraw at any time without consequences.

## 3. Results

An effective response rate of 85.6% was achieved, yielding 510 valid questionnaires obtained. The results of Harman’s single‐factor test indicated that 14 factors had eigenvalues greater than 1. The first factor explained 36.3% of the total variance, which was below the critical threshold of 40%, suggesting that no severe common method bias was present in this study [18].

### 3.1. Sample Characteristics

Most of the participating nurses were female (95.5%), aged 30–40 years (51.0%), and married (77.5%). More detailed demographic characteristics are presented in Table 1.

**TABLE 1:** Characteristics of participants and difference in occupational resilience (*n* = 510).

Characteristics	*n* (%)	CMI		
		Mean (SD)	*t*/*F*	*p*
Gender			−1.322	0.187
Male	23 (4.5)	95.13 (17.33)		
Female	487 (95.5)	99.39 (14.99)		
Age (years old)			7.928	**< 0.001**
< 30	132 (25.9)	95.82 (14.54)		
30–40	260 (51.0)	98.58 (15.05)		
41–50	97 (19.0)	103.37 (14.99)		
> 50	21 (4.1)	108.76 (12.41)		
Marital status			9.903	**< 0.001**
Married	395 (77.5)	100.75 (15.12)		
Unmarried	109 (21.4)	93.60 (13.84)		
Divorced/widowed	6 (1.2)	99.00 (15.05)		
Education			0.102	0.903
Associate degree or lower	76 (14.9)	99.12 (15.80)		
Undergraduate	415 (81.4)	99.28 (14.98)		
Master’s degree or above	19 (3.7)	97.68 (15.74)		
Professional title			14.125	**< 0.001**
Junior professional	219 (42.9)	95.61 (15.53)		
Intermediate professional	251 (49.2)	101.09 (14.29)		
Senior professional	40 (7.8)	106.98 (12.84)		
Occupational role			−3.785	**< 0.001**
Clinical nurses	444 (87.1)	98.23 (15.13)		
Nurse managers	66 (12.9)	105.68 (13.33)		
AI‐related training participation			17.145	**< 0.001**
Yes	136 (26.7)	103.72 (13.73)		
No	374 (73.3)	97.55 (15.27)		

*Note:* The bold values indicate statistically significant results (*p* < 0.05). CMI, Career Motivation Inventory Scale.

### 3.2. Differences in Occupational Resilience According to Nurses’ Characteristics

The results showed statistically significant differences in occupational resilience across age groups, marital status, professional titles, occupational roles, and AI‐related training participation (*p* < 0.05) (Table 1).

### 3.3. Correlation Analysis Between Variables

Descriptive statistics for all study variables are detailed in Table 2. Correlation analyses confirmed significant associations among all core variables, providing preliminary support for the proposed mediation pathways (Table 3).

**TABLE 2:** Scale scores and correlation coefficients of study variables (*n* = 510).

Variables	Mean (SD)	1	2	3	4
1. AI literacy	62.28 (9.15)	1			
2. Psychological capital	108.93 (14.73)	0.356^∗∗^	1		
3. Burnout	55.11 (8.07)	−0.130^∗^	−0.408^∗∗^	1	
4. Occupational resilience	99.20 (15.11)	0.351^∗∗^	0.777^∗∗^	−0.457^∗∗^	1

Abbreviation: AI literacy, Artificial Intelligence Literacy.

^∗^ *p* < 0.05.

^∗∗^ *p* < 0.001.

**TABLE 3:** Regression analysis of variable relationships in the mediation model (*n* = 510).

	Psychological capital				Burnout				Occupational resilience			
	*B*	*β*	SE	*t*	*B*	*β*	SE	*t*	*B*	*β*	SE	*t*
Constant	69.679		4.976	14.002^∗∗^	80.968		3.230	25.067^∗∗^	33.013		6.051	5.456^∗∗^
AI literacy	0.558	0.347	0.068	8.199^∗∗^	−0.007	−0.007	0.040	−0.165	0.138	0.083	0.050	2.773^∗^
Psychological capital					−0.212	−0.386	0.025	−8.598^∗∗^	0.676	0.659	0.033	20.570^∗∗^
Burnout									−0.303	−0.162	0.056	−5.422^∗∗^
Age 30–40 (years old)	0.547	0.019	2.088	0.262	0.784	0.049	1.148	0.682	−1.995	−0.066	−1.394	−1.394
Age 41–50 (years old)	4.15	0.111	2.717	1.527	0.117	0.006	1.498	0.078	−2.355	−0.061	1.866	−1.262
Age > 50 (years old)	5.801	0.078	3.889	1.492	−0.166	−0.004	2.143	−0.078	0.075	0.010	2.67	0.281
Married	2.115	0.017	1.871	1.199	−1.146	−0.111	1.034	−1.076	2.177	0.060	1.294	1.683
Divorced/widowed	1.161	0.009	5.731	0.203	−2.398	−0.199	2.152	−1.347	1.906	0.014	3.948	0.483
Intermediate professional title	2.25	0.076	1.706	1.319	−0.292	−0.018	0.940	−0.311	1.691	0.056	1.171	1.444
Senior professional title	1.593	0.029	3.293	0.484	−0.581	−0.019	1.812	−0.321	3.014	0.054	2.257	1.335
Nurse managers	0.928	0.021	2.189	0.424	−0.791	−0.033	1.204	−0.657	−0.019	0.000	1.501	−0.013
No AI‐related training participation	−1.838	−0.055	1.407	−1.306	−1.045	−0.057	0.775	−1.349	−0.93	−0.027	0.967	−0.962
*F*	11.997^∗∗^				10,543^∗∗^				74.282^∗∗^			
*R* ^2^	0.194				0.189				0.642			

Abbreviation: AI literacy, Artificial Intelligence Literacy.

^∗^ *p* < 0.05.

^∗∗^ *p* < 0.001.

### 3.4. Chain Mediation Effects of Psychological Capital and Burnout on the Relationship Between AI Literacy and Occupational Resilience

Before constructing the regression model, multicollinearity diagnostics were performed on all variables that showed statistical significance in the preceding univariate analyses. The variance inflation factor (VIF) values of all variables ranged from 1.104 to 3.268, all below 5, indicating no significant multicollinearity. All basic assumptions required for serial mediation analysis were verified. The linear relationships among core variables, normal distribution of residuals, and homoscedasticity were all satisfied. The overall model demonstrated good robustness and reliable results.

**FIGURE 1:**
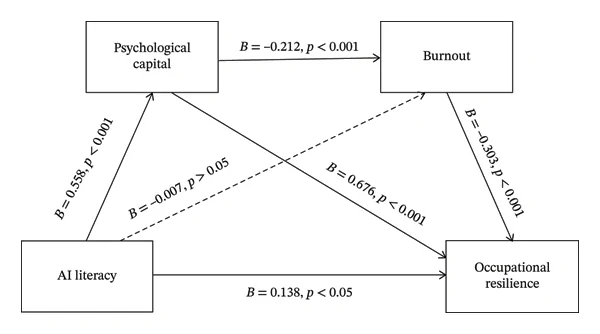
The chain mediation model of AI literacy and occupational resilience through psychological capital and burnout.

Regression results (Table 3 and Figure 1) showed that AI literacy positively predicted psychological capital, which in turn negatively predicted burnout and positively predicted occupational resilience. Burnout negatively predicted occupational resilience. With both mediators included, AI literacy remained a significant direct predictor of occupational resilience, indicating partial mediation. Significant covariates (i.e., age, marital status, professional title, occupational role, and AI‐related training participation) from the univariate analyses were controlled for in the mediation model.

Bootstrap mediation analysis (Table 4) further decomposed the effects of AI literacy on occupational resilience. The total effect (effect = 0.552, 95% CI [0.416, 0.689]), direct effect (effect = 0.138, 95% CI [0.040, 0.235]), and total indirect effect (effect = 0.415, 95% CI [0.309, 0.526]) were all significant. Two valid indirect pathways were identified. The dominant single mediation pathway via psychological capital accounted for 68.3% of the total effect. A small but significant sequential chain mediation pathway via psychological capital and burnout contributed only 0.7% of the total effect. The indirect pathway through burnout alone was not statistically significant.

**TABLE 4:** Chain mediation analysis of AI literacy and occupational resilience (*n* = 510).

Effect	Pathways	Effect	Boot SE	Boot		Effect proportion (%)
				LLCI	ULCI	
Total effect		0.552	0.069	0.416	0.689	100
Direct effect	AI literacy ⟶ occupational resilience	0.138	0.050	0.040	0.235	25.0
Total indirect effect		0.415	0.056	0.309	0.526	75.2
Indirect effect 1	AI literacy ⟶ psychological capital ⟶ occupational resilience	0.377	0.049	0.283	0.475	68.3
Indirect effect 2	AI literacy ⟶ burnout ⟶ occupational resilience	0.002	0.012	−0.023	0.026	0.04
Indirect effect 3	AI literacy ⟶ psychological capital ⟶ burnout ⟶ occupational resilience	0.036	0.009	0.019	0.057	0.7

Abbreviation: AI literacy, Artificial Intelligence Literacy.

## 4. Discussion

To the best of our knowledge, this study is the first to examine the relationship between AI literacy and occupational resilience and to test the chain mediating role of psychological capital and burnout among clinical nurses.

Participants showed a moderate‐to‐high level of occupational resilience, partly because 57% of the sample held junior or higher professional titles. With extensive work experience and efficient practice strategies, these nurses were better able to handle diverse tasks and challenges, which in turn enhanced their occupational resilience. The mean AI literacy score was similar to that reported among nurses in Hunan Province (63.86 ± 8.91) [19], indicating considerable room for improvement in AI literacy among Chinese nurses.

Correlation analysis revealed a significant negative association between psychological capital and burnout, consistent with a prior meta‐analysis [20]. This finding suggests that higher psychological capital may help protect nurses from burnout. As a positive psychological resource, psychological capital may enable individuals to cope with adverse and stressful work events, thereby reducing burnout. Similarly, higher AI literacy was associated with higher psychological capital and lower burnout, aligning with previous research [8].

However, it should be emphasized that these correlations should not be overinterpreted as direct causal links. The relationship between AI literacy and burnout is shaped by contextual constraints, such as hospital digital infrastructure and resource allocation for AI training and implementation. Moreover, a reverse mechanism cannot be ruled out: Nurses with mild burnout may have more energy and motivation to learn and adopt intelligent nursing tools, offering an alternative explanation for the observed correlation.

Furthermore, we found a positive association between psychological capital and occupational resilience, supported by qualitative evidence [21]. Despite their correlation, psychological capital and occupational resilience are distinct constructs. Psychological capital refers to an individual’s positive psychological resources—including self‐efficacy, hope, optimism, and psychological resilience—developed through personal growth [22]. Occupational resilience, by contrast, refers specifically to the ability to withstand work‐related stress and adversity [1], emphasizing contextual coping rather than broad resource accumulation. We also found that lower burnout was significantly associated with higher occupational resilience, consistent with earlier studies on the determinants of occupational resilience [23].

Further analysis indicated a direct positive effect of AI literacy on nurses’ occupational resilience. As a professional skill in the digital era, AI literacy enables highly competent nurses to use intelligent nursing tools proficiently—optimizing workflows, reducing repetitive workloads, and enhancing clinical competence [5]. Improved professional competence directly strengthens nurses’ psychological and behavioral capacities to cope with occupational stress and challenges, thereby positively shaping their occupational resilience. Demonstrating this direct effect clarifies the value of cultivating AI literacy for developing occupational resilience and suggests that providing AI skill training may be an effective strategy to enhance nurses’ occupational resilience.

We identified two mediating pathways: a dominant single‐mediator pathway via psychological capital and a supplementary chain‐mediator pathway via psychological capital and burnout.

First, psychological capital played an independent and pivotal mediating role, accounting for 68.3% of the total effect. Based on the COR theory, AI literacy can be viewed as a digital professional resource for nurses. The accumulation of such skills not only directly improves work capacity [24] but also transforms into internal psychological resources over time. Nurses with higher AI literacy may experience greater professional accomplishment and self‐efficacy in digital nursing practice, fostering core dimensions of psychological capital, such as hope, optimism, and tenacity. As a key personal resource for coping with occupational stress, psychological capital serves as a foundational driver of occupational resilience [25]. A study exploring the mediating role of resilience between psychological capital and innovative behavior among nurses showed that psychological capital had a direct, significant, and positive predictive effect on resilience (*β* = 0.310) [26], whereas the same pathway in the present study yielded a larger coefficient (0.659). This difference suggests that psychological capital exerts a stronger influence on nurses’ occupational resilience when AI is applied in clinical practice, further confirming its essential position in the mediation model.

Second, the sequential mediation pathway via psychological capital and burnout reached statistical significance but accounted for only 0.7% of the total effect, with a small effect size, representing a secondary mechanism. From the JD‐R perspective, psychological capital buffers the impact of work‐related demands, reducing the risk of burnout—a critical health impairment process triggered by prolonged stress. Aligning with COR theory, enhanced psychological capital helps nurses conserve and replenish emotional resources, mitigating burnout manifestations, such as emotional exhaustion and depersonalization. Reduced burnout, in turn, lowers occupational psychological strain, enabling nurses to maintain positive work engagement and reinforcing their occupational resilience [9]. While the practical significance of this small effect should be interpreted cautiously, it offers a nuanced perspective for nursing management: Managers may combine psychological capital cultivation with burnout intervention in nursing support work.

Notably, despite a strong and significant correlation between AI literacy and burnout, this study did not support a simple mediating path from AI literacy to occupational resilience through burnout alone. This indicates that psychological capital is a necessary mediator for AI literacy to influence burnout and that such a correlation cannot be directly transmitted to occupational resilience. According to COR theory, this nonsignificant mediation implies that burnout alone—as a single indicator of resource loss—is insufficient to explain how AI literacy shapes occupational resilience. The reasons are as follows: First, correlation does not equate to an effective mediating path. Correlation only reflects the covariation, whereas valid mediation requires independent variable to significantly affect the dependent variable through the mediator under strict statistical criteria. Second, psychological capital is a necessary bridge for AI literacy to reduce burnout. As a professional skill resource, AI literacy cannot directly alleviate burnout. It must first be converted into internal psychological capital, which then improves nurses’ psychological adjustment and indirectly mitigates burnout. Without this transformation, the negative correlation between AI literacy and burnout cannot effectively promote occupational resilience.

## 5. Strengths and Limitations

One strength of this study is its multicenter design across diverse hospital levels and regions, which improved sample diversity. Furthermore, all variables were assessed using validated scales that demonstrated good reliability in the present study.

Several limitations should be acknowledged. First, because this is a cross‐sectional study, it can only identify correlations rather than causal relationships among variables. Without longitudinal tracking data, the temporal order of variable changes cannot be established, which reduces the certainty of inferred mediating pathways.

Second, all measurements were collected via self‐report scales. Although Harman’s single‐factor test was performed to examine common method bias, this method can only serve as a preliminary check and cannot completely eliminate such systematic bias. Potential response tendencies and subjective reporting bias may still affect the authenticity of data. Future studies could adopt multisource evaluation methods, combine objective behavioral indicators with peer or supervisor ratings, and use staggered measurement time points to better control for common method bias.

Third, this study used convenience sampling, which may introduce potential selection bias. All participants were recruited from urban settings; therefore, the findings may not fully reflect the actual situation of nurses working in rural medical institutions. Moreover, given cross‐country differences in working environments, the results may not be directly generalized to medical institutions in other countries.

Fourth, although multicollinearity diagnostics were conducted in advance and no severe multicollinearity was detected among the variables, some study constructs have similar connotations and overlapping conceptual boundaries, which may weaken the discriminant validity of the relevant indicators.

## 6. Conclusion

This study clarifies the mechanisms underlying the relationship between nurses’ AI literacy and occupational resilience. The findings indicate that AI literacy enhances occupational resilience through the chain mediating effects of psychological capital and burnout, with psychological capital serving as the core mediator. These results support COR theory and extend research on occupational resilience mechanisms in digital nursing settings, providing empirical evidence for AI literacy training and psychological capital interventions in the nursing field.

## 7. Implications for Nursing Management

At the individual level, hierarchical AI training programs for nurses are recommended. Basic digital literacy and safety training should be arranged for junior nurses to establish fundamental AI application awareness. Senior nurses should receive scenario‐based practical training focusing on daily clinical AI functions, including intelligent documentation, clinical decision support, and follow‐up management. It is also necessary to demonstrate the practical value of AI tools in daily nursing procedures to improve nurses’ acceptance and willingness to use relevant technologies. We suggest using the validated PCQ‐24 scale for quarterly assessments of nurses’ psychological capital, with additional evaluations arranged upon recruitment and job rotation. Managers can provide one‐on‐one guidance and group counseling to nurses with low psychological capital to relieve stress. For those with moderate psychological capital, regular experience‐sharing meetings and brief mindfulness activities should be held to maintain their psychological stability. Moreover, given the mediating role of burnout in the chain, managers should implement regular burnout screening and offer targeted interventions—such as workload redistribution, peer support groups, or mindfulness‐based stress reduction programs—to interrupt the resource depletion process.

From organizational and managerial perspective, healthcare institutions are recommended to implement advanced AI‐enabled nursing technologies (e.g., AI‐based nursing systems). Digital infrastructure should be upgraded by deploying state‐of‐the‐art AI algorithms and building dedicated hospital computing platforms to support stable AI operation and efficient clinical data processing. Meanwhile, standardized technical access protocols and intelligent nursing application guidelines should be established to ensure safe, efficient, and consistent use of AI tools across clinical settings.

## Author Contributions

Qingzhu Qin was responsible for the study design, acquisition of data, and drafting the manuscript. Wenjing Sun checked the statistical methods and interpretation of results in this study. Hengyu Hu was responsible for the acquisition of data. Yixiu Du and Cancan Chen made equal contribution to the conception of the work, the interpretation of the data, and revised the manuscript.

## Funding

No funding was received for conducting this study.

## Conflicts of Interest

The authors declare no conflicts of interest.

## Data Availability

The data that support the findings of this study are available from the corresponding author upon reasonable request.
